# Autonomic and Enteric Nervous System Dysfunction May Play a Role in Hyperemesis Gravidarum

**DOI:** 10.14740/gr632w

**Published:** 2015-02-14

**Authors:** Joy D. Hughes, Neeta G. Nayak, Naeem Aslam, Hani Rashed, Sergio Cardoso, Babajide Familoni, James G. Karas, David C. Shaver, Robert S. Egerman, Alan Wallstedt, Caroline A. Riely, Thomas L. Abell

**Affiliations:** a Mayo Clinic, Rochester, MN, USA; b Departments of Internal Medicine (Gastroenterology Division) and Obstetrics/Gynecology, and the Autonomic Function Lab, University of Tennessee Bowld Hospital, The University of Tennessee, Memphis, TN, USA; c Department of Electrical Engineering, University of Memphis, Memphis, TN, USA; d Division of Gastroenterology, Hepatology, and Nutrition, University of Louisville, Louisville, KY, USA

**Keywords:** Nausea and vomiting, Hyperemesis gravidarum, Pregnancy, Weight loss, Autonomic nervous system, Enteric nervous system

## Abstract

**Background:**

Nausea and vomiting, seen in 70-85% of all pregnancies, becomes intractable in hyperemesis gravidarum (HG). We aimed to investigate the relationship between HG and autonomic nervous system functioning and gastric electrical activity.

**Methods:**

Twenty-seven pregnant patients, 21 with HG and six normal, were studied with sympathetic adrenergic; percent vasoconstriction (%VC) and postural adjustment ratio (PAR); parasympathetic vagal cholinergic functions by R-to-R intervals (RRIs), a total autonomic score; and enteric nervous system measured by electrogastrography (EGG).

**Results:**

Significant differences were found in parasympathetic measures (RRI for HG 29.98 ± 2.95 vs. control 40.91 ± 2.38, P < 0.05); sympathetic PAR was significantly lower in patients (PAR for HG 24.5 ± 5.0 vs. 67.6 ± 11.4 for controls, P < 0.01); mean total autonomic score was significantly lower in HG (131.75 ± 9.61 vs. 196.87 ± 12.8, P < 0.05). EGG results were borderline different (normal < 3.3, HG 3.4 vs. controls 3.0, P = 0.07).

**Conclusion:**

Autonomic and enteric nervous system dysfunction may play a role in the pathophysiology of HG.

## Introduction

Hyperemesis gravidarum (HG) is the severe presentation of “morning sickness”, with no clear delineation between the expected nausea and vomiting of early pregnancy and HG. The nausea and vomiting associated with pregnancy typically presents by 9 - 10 weeks of gestation, peaks at 11 - 13 weeks, and then resolves by 12 - 14 weeks; in 1-10% of pregnancies, symptoms may continue in 50% of weeks beyond 20 - 22 weeks of gestation [1-4]. Hyperemesis has been defined using the following criteria: persistent vomiting, weight loss exceeding 5% of pre-pregnancy body weight, and ketonuria [5].

Documentation of vomiting associated with early pregnancy appeared in the medical literature in the 17th century [6]. Medical practitioners of the time proposed treatment modalities such as fasting, termination of pregnancy, and donation of blood from the husband. Overall prognosis was poor, and death due to dehydration and electrolyte imbalance was common [7]. Today, patients with HG frequently present with ketosis and electrolyte derangements such as hypokalemia and metabolic alkalosis due to intractable vomiting [3]. Other lab abnormalities may include elevated blood urea nitrogen, abnormal liver enzymes, and an increase in hematocrit indicating hemoconcentration due to plasma volume depletion [2, 8, 9].

Studies have shown an association between HG and high body weight, nulliparity, familial predisposition, and multiple gestations. Several theories concerning the etiology of HG have been proposed, including thyroid abnormalities, reproductive hormones, liver, gastric electrical activity, lipids, and psychological state, as well as nutritional [2]. In addition, some colleagues have observed autonomic nervous system abnormalities, but sufficient data supporting autonomic abnormality as an underlying etiology for HG are lacking. One study reports biphasic changes in autonomic activity in pregnancy, with a shift of the autonomic nervous system towards a lower sympathetic and higher vagal modulation in the first trimester, and then another shift towards a higher sympathetic and lower vagal modulation in the third [10-12]. Other studies support this hypothesis of autonomic imbalance [13]. Additionally, studies of persistent nausea and vomiting in diabetic gastroparesis have shown blunted adrenergic responses and electrogastrogram (EGG) abnormalities [14]. We aimed to investigate the relationship between HG and autonomic nervous system functioning, hypothesizing that HG may be related to imbalance in autonomic nervous system functioning and gastric electrical activity and that autonomic function testing (AFT) is useful in predicting HG in pregnant women with nausea and vomiting.

The objective of our study was to prospectively assess abnormalities in autonomic functions in a group of patients with HG. We also investigated association between autonomic dysfunction and anomalous thyroid and liver function tests.

## Materials and Methods

### Patient population

We studied 21 post-rehydration HG patients hospitalized for ketosis based on clinical and laboratory parameters and six healthy pregnant control patients. There were no statistical differences between groups in mean age (23 vs. 27 years), weight (140 vs. 131 lbs), or gestational age (13.6 vs. 15.1 weeks). The HG patients were largely multiparous (14 out of 21 patients (66%)) while all but one of the normal controls was multiparous (83%).

### AFTs

To assess autonomic nervous system functioning, measures of sympathetic adrenergic function (SAF), vagal cholinergic function (VCF), and sympathetic cholinergic function (SCF) were performed. Two measures of SAF were performed, each by utilizing capillary photoplethysmography. Percent vasoconstriction (%VC) in response to cold stress is a measure of the change in capillary pulse amplitude caused by reflex vasoconstriction and is expressed as percentage of change from baseline. Blood flow was measured via capillary pulse amplitude in the left hand with infrared photoplethysmography while the right hand was simultaneously inserted into water maintained at 14 °C for 60 s. Postural adjustment ratio (PAR), which is the ratio in response to raising and lowering the arm, was assessed by measuring blood flow in one hand while that hand was raised, lowered, and held level. PAR was expressed as a ratio of a reflex barostatic response from the baseline.

VCF was measured as variability in pulse rate during deep respiration, with respiratory peaks designated as R and using the R-R interval (RRI) on the EKG with a continuous electrocardiogram strip taken while the patient breathed maximally. The time intervals between respiratory peaks were calculated and a vagal response (expressed as a percentage) was then assessed as RRI using the following formula: RRI = ((inspiration time - expiration time)/expiration time) × 100.

SCF was measured by resting skin temperature in degrees centigrade. Total autonomic score (TAS = VC + PAR + RRI) was also calculated as previously reported [10].

Resting electrogastrography (EGG) in cycles/min was recorded as a non-invasive measure of the enteric nervous system [11]. Resting gastric electrical activity was studied by recording a resting cutaneous tracing for 30 min, which we have previously correlated well with a 4-h fasting and fed EGG [12]. Frequency is reported in cycles/min (normal is < 3.3 cycles/min). Three investigators evaluated tracings blinded for identifiers. All patient tests were conducted in the same manner after initial skin temperature and baseline measurements showed stability. No patients presented clinical symptoms of dehydration when studied.

Autonomic function data were compared with data from a group of 21 healthy pregnant women and 49 normal volunteers (mean age 19.5 ± 0.95 years). All three AFT measures were evaluated by sex variable (male or female) in the normal volunteer group, and no significant differences were found between male and female patients in AFT measures. Values for thyroid and liver function were also compared for normal controls.

### Statistical analyses

To compare autonomic measures of patients with those of controls, AFT and EGG were compared by unpaired *t*-tests results between HG patients and controls and by Spearman correlation coefficients within the HG and the control groups and within the normal and abnormal EGG subgroups. Statistical size calculations were based on previous work with diabetes mellitus patients and similar symptoms. All *t*-tests were two-tailed and statistical significance for *t*-tests was designated P ≤ 0.05 level using a Bonferroni correction factor for multiple comparisons. All data analyses were conducted via the CLINFO software package (Bolt, Beranek, and Newman, Boston, MA, USA) in conjunction with Statistical Analysis Systems (SAS Institute, Research Triangle, NC, USA) on a VAX 11/750 mainframe located at the clinical research center, or by PC!Info, a DOS version of CLINFO. This review was approved by the University of Tennessee-Memphis Institutional Review Board.

## Results

**Figure 1:**
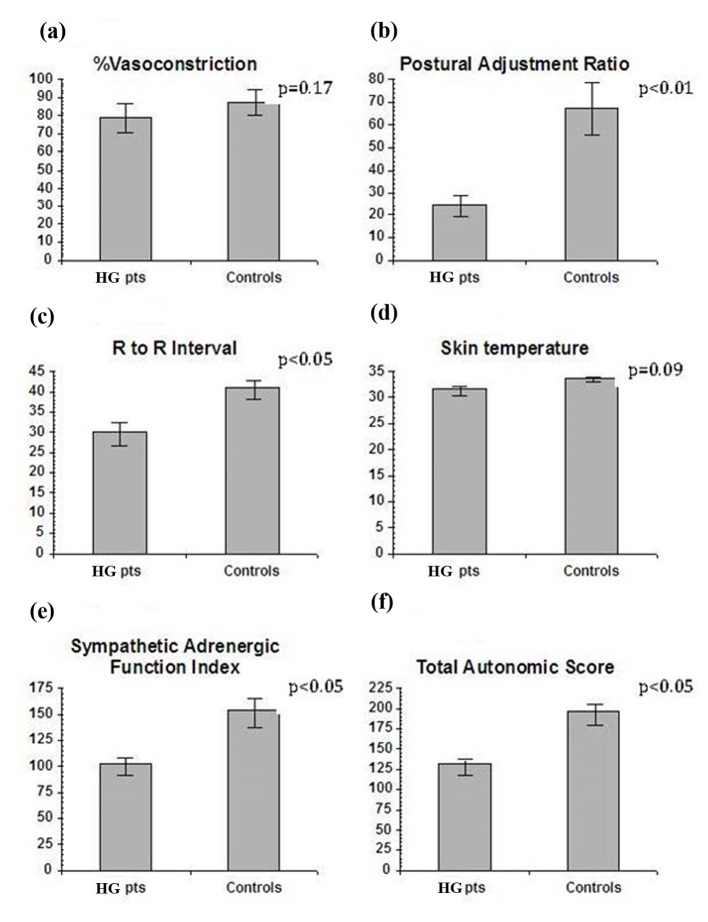
(a) The sympathetic measure of vasoconstriction to cold was impaired in HG vs. pregnant controls but the results were not statistically significant. (b) Postural adjustment ratio, a sympathetic adrenergic measure, was significantly lower in HG vs. pregnant controls. (c) R to R, the vagal cholinergic measure, was significantly less in HG vs. pregnant controls. (d) Skin temperature, a mixed measure, was lower in HG, but of borderline significance. (e) Sympathetic adrenergic function index, the sum of VC + PAR, was significantly impaired in the HG vs. pregnant controls. (f) Total autonomic score, a global measure, was significantly impaired in HG vs. pregnant controls.

Measures of autonomic functions (VC, PAR and RRI) were obtained in all 21 patients and six controls. The sympathetic adrenergic measure of %VC did not show statistical difference in the HG group compared to pregnant controls (mean 79.4 ± 4.2 vs. 87.2 ± 3.6, P = 0.17) (Fig. 1a). However, the sympathetic adrenergic measure PAR was significantly lower in HG patients than in the control group (mean 24.5 ± 5.0 vs. 67.6 ± 11.4, P < 0.01) (Fig. 1b). Likewise, the vagal cholinergic measure of RRI was significantly lower in the HG group than the controls (mean 29.98 ± 2.95 vs. 40.91 ± 2.38, P < 0.05) (Fig. 1c). Skin temperature, a sympathetic cholinergic measure, was low in patients as compared to controls (mean 31.6 ± 0.8 vs. 33.8 ± 0.5, P = 0.09), but the results did not reach statistical significance (Fig. 1d). Significant differences were also found in sympathetic adrenergic function index (SAFI = VC + PAR) (HG group mean 103.4 ± 9.4 vs. control group mean 154.9 ± 14.3, P < 0.05, normal) (Fig. 1e). Total autonomic score (TAS = VC + PAR + RRI) was also lower in HG patients (HG 131.75 ± 9.61 vs. control 196.87 ± 12.8, P < 0.05) (Fig. 1f). EGG results revealed that the control group had a normal EGG frequency at 3.0 cycles with a range of 2.9 - 3.2 cycles per minute, while HG patients had a higher mean of 3.4 cycles per minute, with borderline significant difference from controls (P = 0.07). However, a subgroup of seven HG patients with EGGs in the abnormal range (> 3.3 cycles/min) showed a correlation between higher EGG frequency and the sympathetic adrenergic measure PAR after ice (r = -0.64, P < 0.10).

Thyroid functions (T3 and T3RU) were found to be decreased in the same patients who had abnormal VC (r = 0.5, P < 0.03) and RRI (r = 0.5, P < 0.021) respectively. Also liver transaminases vs. SGOT and SGPT were decreased in those with an abnormal PAR (r = -0.5, P < 0.013) and RRI (r = 0.7, P < 0.001).

## Discussion

Using standardized and well-characterized non-invasive methods, we prospectively assessed a well-defined group of patients who met the criteria for HG. We found that patients with hyperemesis have blunted autonomic nervous system measures when compared with healthy pregnant controls. Similar blunting of response has been reported previously in patients with diabetes mellitus [15]. These findings suggest the presence of altered autonomic neuro-systemic function may be associated with the patients’ symptomatic nausea and vomiting. The same patients who had abnormalities in their thyroid and liver functions also had abnormal AFT results.

Several aspects of AFT methodology need to be mentioned. First, the patient’s level of hydration can affect autonomic testing results [16]. However, in this study all HG patients were clinically rehydrated. While AFT measures have traditionally been related to the study of cardiovascular autonomic responses, here they were applied to gastrointestinal function. However, a number of studies have shown that general autonomic responses may reflect gastrointestinal function [17]. While the data may be primarily descriptive, we believe that these findings may provide new clues to the mechanism of HG. Our findings of EGG abnormalities showed a connection of this enteric measure with the SAF.

### Conclusion

HG is a poorly understood disorder with little certainty about its pathogenesis. Although many theories have been proposed to understand HG, including thyroid or liver dysfunction and psychological or behavioral abnormalities, none has been defined as the primary cause. In our study, we found significant blunting of autonomic and enteric responses in HG patients as compared to pregnant controls. We conclude that the autonomic and enteric nervous systems may play a role in modulating primary symptoms in HG, and may serve as a model for further research in patients with hyperemesis.
